# Poly (Aryl Amino Ketone/Sulfones) with Obvious Electrochromic Effect Prepared by One-Step Low-Cost and Facile Synthesis

**DOI:** 10.3390/molecules28145297

**Published:** 2023-07-09

**Authors:** Songrui Jia, Zhen Xing, Qilin Wang, Shiwei Wang, Zheng Chen

**Affiliations:** 1 Key of High Performance Plastics, Ministry of Education, National & Local Joint Engineering Laboratory for Synthesis Technology of High Performance Polymer, College of Chemistry, Jilin University, Changchun 130012, China; jiasr21@mails.jlu.edu.cn (S.J.); xingzhen21@mails.jlu.edu.cn (Z.X.); wangql22@mails.jlu.edu.cn (Q.W.); 2 School of Chemical Engineering, Changchun University of Technology, Changchun 130012, China

**Keywords:** electrochromism, C-N coupling, PAASs/PAAKs

## Abstract

High-performance donor-acceptor (D-A) polymers, as an important class of electrochromic (EC) materials, have attracted extensive attention. In this paper, a series of novel poly (aryl amino ketone) (PAAK) and poly (aryl amino sulfone) (PAAS) type high-performance polymers (HPP) with electrochromism were prepared by a simple C-N coupling reaction and were coated on an indium tin oxide (ITO) substrate as EC films. All four polymers were prepared by a nucleophilic substitution reaction using commercially purchased amine monomers with difluoride sulfone/ketone using potassium carbonate as a catalyst. A series of tests were performed to compare and analyze the effects of the different electron-withdrawing abilities of sulfone and carbonyl groups, and the different conjugation lengths of these two TPA structures were connected to the EC properties of the polymer. The different phenyl or biphenyl of the two TPA structures mainly affected the oxidation potential of the polymer, while the sulfone group and the carbonyl group, with a different electron absorption ability, had a greater influence on the energy band and cyclic stability. The optical contrast of PAAS−BT at 850 nm was up to 58% and maintained 450 cycles, indicating that this series of materials had a broad application prospect waiting for further research. In addition to the performance, the raw materials used in this work could be directly and commercially purchased for a low price; the two aniline monomers were priced at about $0.43 /g and $0.15 /g, respectively. This method significantly reduces the cost and provides a new idea for subsequent large-scale production and practical applications.

## 1. Introduction

Electrochromic (EC) materials can shift the absorption wavelength and change the absorption intensity after the application of oxidation or a reduction in voltage. Due to its reversible change and regulation under different voltages, EC materials are widely used in smart Windows [1], electronic labels [2], color-changing stickers [3], electronic ink [4], electronic paper [5], and smart glasses [6]. Compared with other inorganic materials and small organic molecules, which can only show a single color change, electrochromic polymers (ECPs) make it easy to obtain various rich colors with low band gaps through structural modification and have been extensively studied [7]. Among the various methods of EC polymer design, it is the most effective and widely used strategy to select the appropriate electron-accepting unit and electron-donating group to form the donor-acceptor (D –A) interacting unit when regulating the electrochemical behavior of a polymer [8]. Currently, a variety of ECPs with desirable properties have been designed and successfully synthesized and can even be used in the commercial production and application of optical devices.

Generally speaking, EC polymers with a lower conjugation degree of the backbone have a lower absorption capacity in their visible wavelength range, and processed film shows a higher transmittance in the neutral state, which can be intuitively observed as a light-colored transparent film. In contrast, high-performance polymers (HPPs) such as polyamides [9], polyimides [10], and poly aryl sulfone/ketone [11,12,13] exhibit thermal stability that is comparable to other conjugated polymer-based EC materials and electroactive groups can be directly introduced into the polymer backbone during the structural design process. Therefore, HPPs with high transmittance and thermal stability are significantly more conducive to optical modulation and easily observed color changes in practical applications than dark polymer materials with a high conjugation.

Triphenylamine and its derivatives are good electron donors and hole transport materials [14] and have been widely used for their incorporation into the acceptor moiety in the backbone of high-performance polymers. For example, in the previous work of our group, a series of D-A high-performance electrochromic polymers composed of diphenyl sulphonyl electron acceptors and triphenylamine-based electron donors were successfully synthesized by a C-N coupling reaction, which had a fast switching speed of close to 4 s and a high cyclic stability of 3900 cycles [15].

In this work, four high-performance D-A type EC polymers, poly (aryl amino ketone)s (PAAKs) and poly (aryl amino sulfone)s (PAASs), were synthesized by a simple C-N coupling reaction. They consisted of two triphenylamine (TPA) groups as electron donors (D) and a sulfone or carbonyl unit as an electron acceptor (A), where the two N atoms were linked by phenyl or biphenyl conjugated bridges of different lengths. The raw materials N, N’ -Diphenyl-*p*-phenylenediamine and N, N’-Diphenylbenzidine were commercially purchased, priced at around $0.43 /g and $0.15 /g, respectively, and could be used for mass production. Here, we compare the effects of sulfone and ketone groups with different electron-absorbing abilities and the conjugate distance between the nitrogen atoms of the two oxidation centers on the electrochemical and electrochromic behavior of EC polymers. The best-performing polymer, PAAS−BT, showed an optical modulation of up to 58% and stable cycling of 450 cycles.

## 2. Results

### 2.1. Design and Synthesis

D-A interacting polymer structures are generally used to reduce the oxidation onset potential of polymers, and conductive polymers can avoid the performance degradation caused by peroxidation thanks to low resistance. For example, in the field of EC, lower oxidation initiation potential can make the polymer undergo obvious color changes under a lower potential stimulation and prolong the cycling stability of color change and the service life of devices [16]. In our previous work, we synthesized a variety of PAAKs and PAASs through a two-step C-N coupling reaction [15,17]. This method could be extended to construct cross-conjugated polymers with various donor and acceptor parts (sulfone and ketone groups become cross-conjugated structures in this type of polymer material).

**Scheme 1:**
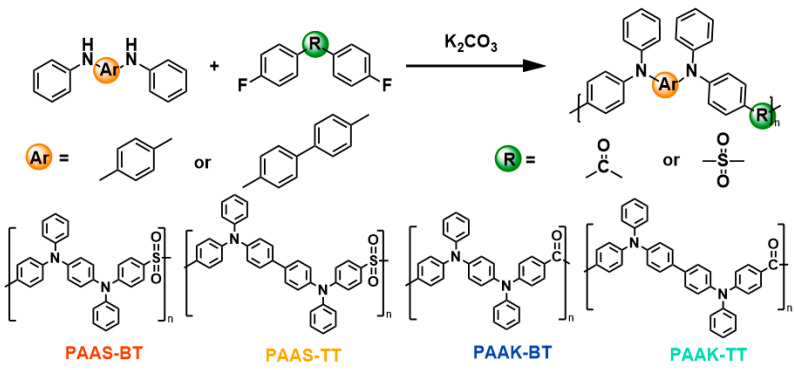
Synthesis of PAAKs/PAASs via one-step C-N coupling-based polycondensation.

In this study, four PAAKs and PAASs were synthesized by a one-step C-N coupling reaction in accordance with the same method, as shown in Scheme 1 and divided into two groups for easy comparison, in which the two N atoms of redox in each group were connected by conjugated units of different lengths, namely the phenyl ring and biphenyl structure. Details about the synthesis and structural characterization are described in detail in the supporting information. 

### 2.2. Basic Characterization

Considering the practical application scenario, the EC polymer needed good solubility. Table S1 shows the results of a qualitative solubility test using 10 mg of the polymer and 1 mL of the solvent. These data indicate that PAASs/PAAKs were soluble in most common solvents, such as methylene chloride, chloroform, dimethyl sulfoxide (DMSO), N, N- Dimethylformamide (DMF), and N-Methylpyrrolidone (NMP). This wide solubility enabled PAASs/PAAKs to be spin-coated or dip-coated for the fabrication of optoelectronic devices. However, it is worth noting that the test condition was only selected as 10 mg/mL, while the solubility of these four polymers exceeded 50 mg/mL in chloroform and also reached 25 mg/mL in NMP: a solvent commonly used in spin-coating processes. Unexpectedly, PAAKs can only partially dissolve in DMF, which is also a solvent with a high boiling point; however, their solubility can still reach 3 mg/mL, which is suitable for the determination of the molecular weight and dispersion by gel permeation chromatography (GPC). As shown in Table 1, all polymers except PAAK−TT had a number-average molecular weight of close to 10^4^ and a relatively concentrated molecular weight distribution. These Pd values were close to one because the polymer underwent multiple extraction processing with a Soxhlet extractor. 

The thermal properties of these polymers were investigated by differential scanning calorimetry (DSC) and thermogravimetric analysis (TGA). The information on their thermal behavior is summarized in Table 1. Figures S3 and S4 show their characteristic DSC and TGA curves. According to the DSC results, the glass transition temperatures (T_g_) of both PAASs were in the range of 195–251 °C. As expected, the T_g_ of the PAAS polymer was higher overall than that of the PAAK polymer due to the larger volume of the sulfone group compared to the carbonyl group, which was more unfavorable for the chain segment motion. Similarly, PAAS−TT with the biphenyl structure had a greater rigidity than PAAS−BT, implying a higher T_g_. However, PAAK−TT exhibited the lowest T_g_, which could be attributed to its lower molecular weight. In contrast to the DSC results, the TGA test (Figure S4) showed that the thermal decomposition temperature T_d5_ of the PAAS polymer was generally lower than that of the PAAK polymer, and this difference reached 80 °C. The thermal decomposition temperatures of all four polymers exceeded 440 °C, meeting the temperature limit of major application scenarios. Interestingly, thermal decomposition temperatures in the air atmosphere were generally 14–17 °C higher than those in the nitrogen atmosphere, possibly due to the TPA structure acting as a nitrogenous flame retardant.

### 2.3. Optical Properties

**Figure 1:**
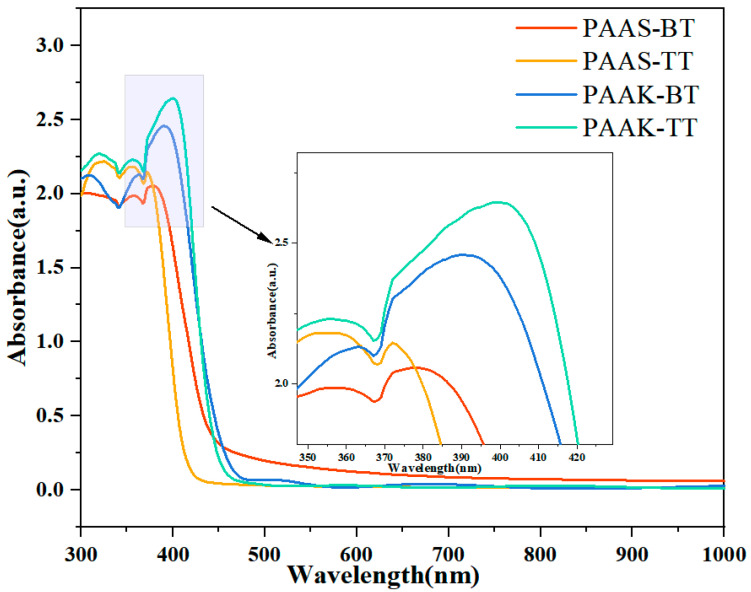
UV-vis absorption spectra of polymers in a film spin-cast on a quartz substrate from the chloroform solution.

The basic optical properties of the four polymers were detected by UV-VIS absorption spectroscopy (Figure 1). All polymers exhibited absorption peaks at 355–365 nm and 373–400 nm, which were characteristic of π–π* transitions and were a result of the presence of the π-conjugated portion of the polymer backbone [18,19]. Since the electron-withdrawing ability and steric hindrance of the carbonyl group were weaker than that of the sulfone group, the absorption peak of PAAK was significantly redshifted compared to PAAS. A similar trend was observed in the solution (Figure S5).

### 2.4. Electrochemical Properties

**Figure 2:**
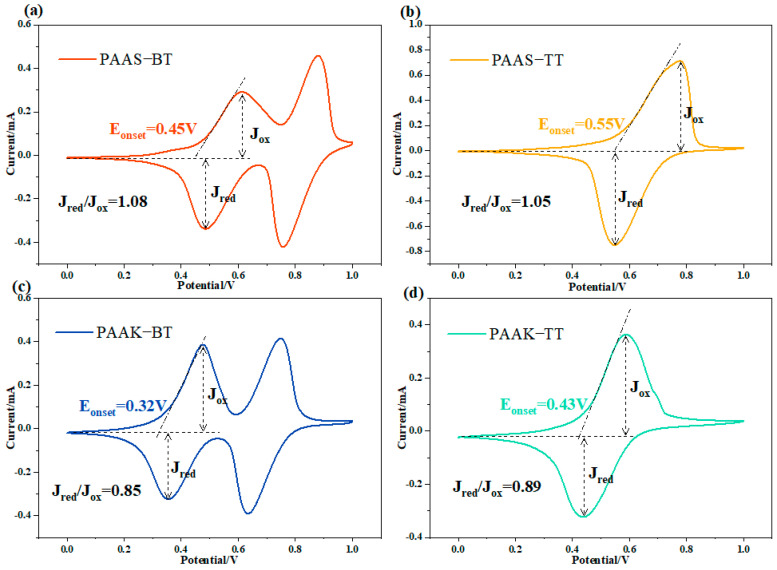
Cyclic voltammograms of (**a**,**b**) the PAAS group and (**c**,**d**) PAAK group films when spin-coated on an ITO glass substrate from the NMP solution at a concentration of 25 mg/mL (Scan rate: 50 mV/s).

The electrochemical properties of four polymers were studied by cyclic voltammetry (CV). The film was cast on the indium tin oxide (ITO) glass substrate and heated in a nitrogen-filled glove box at 150 °C for 8 h to remove the solvent. Then, ITO glass coated with these films was used as the working electrode, as well as the 0.01 M Ag/AgNO_3_ non-hydroelectric electrode and Pt wire, which were selected as the reference electrode and the counter electrode, respectively. The electrolyte is a solution to tetra-*n*-butylammonium perchlorate (TBAP) in dried acetonitrile with a concentration of 0.1 M. The inferred oxidation potentials are listed in Table 2, and the recorded cyclic voltammograms are shown in Figure 2. It is worth noting that polymer films prepared by spin-coating with different solutions could have certain effects on the CV curve (Figure S6). It was obvious that when the concentration of four polymer solutions increased from 10 mg/mL to 25 mg/mL, the peak current of oxidation and reduction all increased while the peak current ratio of PAAK was far from one, indicating that other reaction processes existed in the reaction and could cause a potential error. In addition, peak-to-peak separation (ΔE_p_) with an increase in the polymer solution concentration was amplified, which meant that more of the polymer attached to the ITO glass required a slower kinetic process. Moreover, the phase morphology of the polymer film also affected the kinetic process of redox and could also affect the onset potential of CV curves.

Figure 2 shows that the E_onset_ of PAAK was overall 0.12 V lower than that of PAAS, and PAAS−BT and PAAK−BT exhibited two distinct sets of redox peaks, representing the redox of two nitrogen centers. However, the peaks of PAAS−TT and PAAK−TT containing the biphenyl structure were so close that only one set of redox peaks was shown, and E_onset_ increased by 0.1 V. This could be explained by the fact that the longer biphenyl structure reduced the coupling of cationic radicals in the first oxidation state N^+^ to the other neutral N within the PAAS−TT and PAAK−TT repeating units, showing electrochemical properties similar to the presence of only one triphenylamine unit [20].

In addition, the CV curve was used to obtain E_onset_ and the internal standard of ferrocene (4.80 V; E_onset_ = 0.1 V), while the energy level of the highest occupied molecular orbital (HOMO) of the polymer was calculated after the comparison. As shown in Table 3, through the tangential analysis of the ultraviolet-visible absorption spectrum of the film, the starting absorption wavelength was obtained, and the band gap of the absorption spectrum (E_g_) was deduced. It was obvious that the E_g_ of PAAK was lower than that of PAAS on the whole. Finally, the lowest unoccupied molecular orbital energy (LUMO) was calculated using the obtained results according to the formula in the table.

In the previous work of our research group, a method was proposed to measure the equilibrium of the electron injection and extraction capacity by comparing the ratio of J_red_/J_ox_, that is, the ratio of the reduction peak’s current density to the oxidation peak’s current density (J_red_/J_ox_) [15]. As shown in Figure 2, the J_red_/J_ox_ of PAAS was closer to 1.0, indicating a more balanced electron injection and extraction phenomenon, which could partly explain the better cyclic stability of PAAS. In addition, PAAS−TT and PAAK−TT containing biphenyl structures exhibited J_red_/J_ox,_ which was closer to 1.0; this could be attributed to the longer conjugated segments of biphenyls that helped stabilize the polarons produced by polymers during redox processes.

### 2.5. Theoretical Analysis

**Figure 3:**
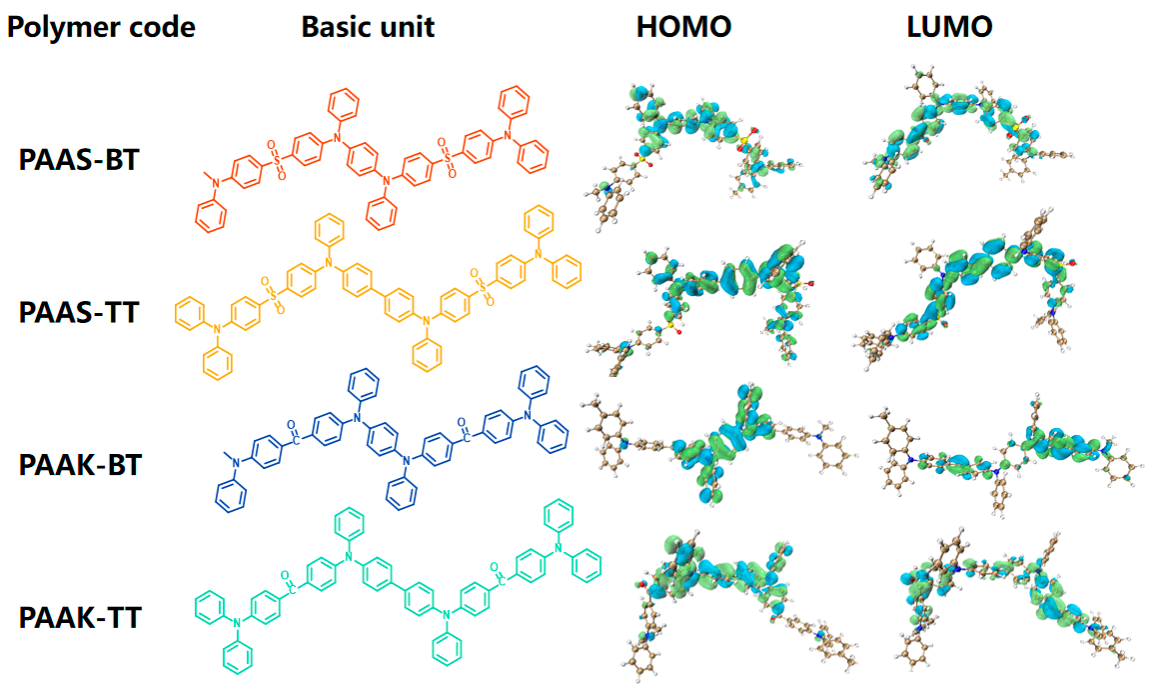
Calculated molecular orbitals of PAAKs/PAASs dimers.

To further understand the trend of the observed optical absorption and energy levels, the intramolecular charge distribution of the fabricated PAAS/PAAK polymers was explored by calculations performed at the TD-DFT/b3lyp/6-311++g (d, p) theoretical level using the Gaussian 16 program [21]. Multiwfn 3.8 software was used for the further analysis of the obtained data, and Visual Molecular Dynamics (VMD) software was used for visualization drawing [22]. Given the complex composition of our polymers, dimers were chosen as model compounds. Figure 3 shows the HOMO and LUMO electron distributions of the model compounds, showing respective energy levels ranging from −4.91 to −5.08 eV and −1.29 to −1.53 eV. The HOMO-LUMO energy data calculated were significantly different from those determined by absorption spectra and cyclic voltammetry (Table 2); however, these observed trends were consistent with actual measurements. These results are summarized in Table 2. It could be seen that there was no significant difference in HOMO and LUMO between PAAS−BT and PAAS−TT, and between PAAK−BT and PAAK−TT; that is, the conjugated length between two N atoms had little effect on the energy level of the polymer. However, compared with PAAK, PAAS exhibited a larger E_g_ on the whole, and the stronger electron absorption capacity of the sulfone group could reduce HOMO/LUMO at the same time, while the smaller steric hindrance of the carbonyl group might be more conducive to the transition of the electron and reduce the band gap. The calculated results show that HOMO was concentrated in the TPA donor skeleton, while LUMO was mainly located around the electron-withdrawing sulfone/carbonyl group.

### 2.6. Spectroelectrochemical Properties

**Figure 4:**
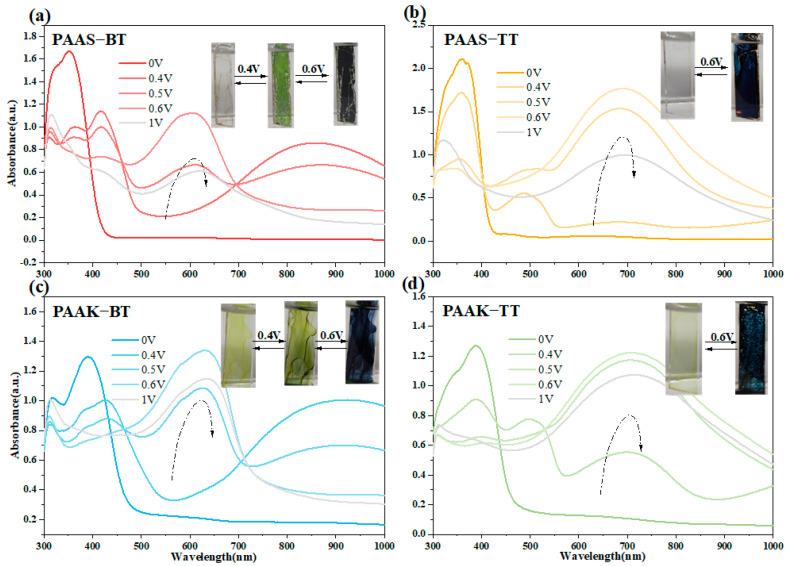
Spectro-electrochemistry of (**a**,**b**) the PAAS group and (**c**,**d**) PAAK group films when spin-cast on the ITO glass substrate from chloroform solution at a concentration of 50 mg/mL.

As shown in Table S1’s solubility test results, the solubility of the polymer in other solvents did not reach the concentration required for the spin-coating of form films. Generally speaking, as a solvent with a high boiling point, NMP demonstrated high viscosity and good solubility for most substances, including polymers, which was conducive to a uniform film coating. However, the absorbance of the polymer film prepared by spin-casting with the NMP solution in our experiment was too low. When the concentration of the NMP solution was 10 mg/mL, the absorbance change was less than 0.1; therefore, the absorption curve was serrated. Even when the concentration of the NMP solution reached 25 mg/mL, the absorbance change in the film was less than 0.2 (Figure S7), which could be due to the polymer solution not fully adhering to the ITO glass and being partially thrown out during the spin-coating process. Therefore, we selected the solvent chloroform with the best solubility to prepare 50 mg/mL of the polymer solution and spin-coated it on ITO glass to measure the spectroelectrochemical properties (Figure 4. In fact, the color-changing polymer films prepared by the spin-coating of these two solutions showed similar spectral absorption variation trends. The electrolyte solution and electrode selection used for the test were the same as those for the CV test described above.

**Figure 5:**
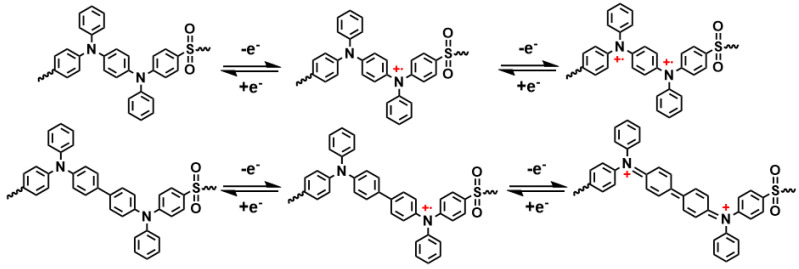
Representation of the oxidized forms of PAAS−BT and PAAS−TT.

For PAAS−BT, there were two N-centers, and thus two oxidation states (Figure 5) and two corresponding color changes could be introduced. When the driving voltage increased to 0.4 V, the absorption intensity at 361 nm began to decline, and two new characteristic absorption peaks appeared simultaneously at 440 nm in the visible region and 865 nm in the near-infrared region. At this time, the color of the film changed from nearly colorless to green. According to the study of Hsiao [19] et al., this change came from the free radical cationic state formed by TPA in the polymer skeleton, and this driving potential was also similar to the first oxidation potential in the cyclic voltammetry test. The wide peaks near the near-infrared were characteristic results of the intervalence charge transfer (IV-CT) excitation associated with the intramolecular electron transfer (ET) from active neutral nitrogen atoms to cationic radical nitrogen centers [23]. As the voltage continued to increase to 0.6 V, the polymer underwent further oxidation, and the absorption peak intensity at 440 nm and near-infrared began to decrease, while the absorption intensity at 600 nm suddenly increased, and the color of this film began to change from green to blue. The second oxidation site was a dictation formed by the two N centers of the tetraphenyl-*p*-phenylenediamine (TPPA) structure [18]. The change between the green and blue colors was completely reversible, and this intense color change could be easily captured by the human eye. Similarly, for PAAK−BT, when the driving voltage increased to 0.4 V, a characteristic absorption at 395 nm decreased, and two new characteristic absorption peaks appeared simultaneously at 446 nm in the visible region and 928 nm in the near-infrared region, showing a reversible change from light yellow and green to dark blue.

However, PAAS−TT and PAAK−TT containing a biphenyl structure only produced sudden absorption peaks at 690 nm and 710 nm, respectively, when the driving voltage rose to 0.6 V, and there was no obvious absorption in the infrared region within 1000 nm. In fact, at a voltage of 0.4 V, the spectral absorption curves of PAAS−TT and PAAK−TT both showed certain absorption enhancements in the infrared region (Figure 5b,d), and we could extrapolate that the absorption peaks of PAAS-TT and PAAK-TT also appeared in the infrared region with a longer wavelength. As mentioned in the section about electrochemical properties, longer biphenyl structures reduced the intramolecular electron transfer of cationic radicals between the two N atoms within PAAS−TT and PAAK−TT repeating units. Therefore, the infrared absorption peak of PAAS−TT and PAAK−TT was redshifted compared with that of PAAS−BT and PAAK−BT.

### 2.7. Electrochromic Properties

**Figure 6:**
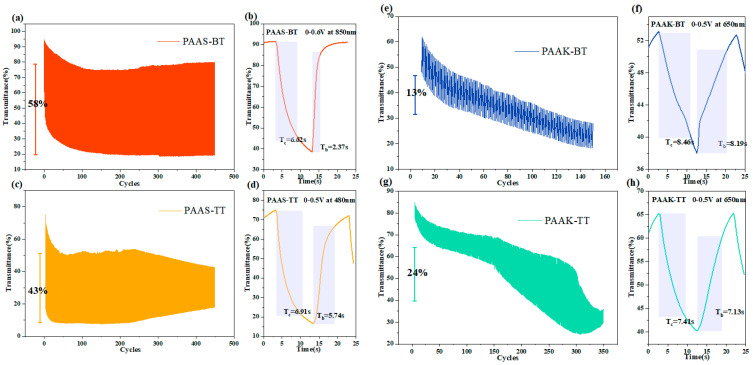
Cyclical stability and switching time of (**a**,**b**) PAAS−BT, (**c**,**d**) PAAS−TT, (**e**,**f**) PAAK−BT and (**g**,**h**) PAAK−TT. Data were acquired using potential step chronoamperometry with a cycle time of 10 s.a–d.

As mentioned in the previous section, EC films prepared with NMP solutions and a polymer concentration of up to 25 mg/mL could only achieve a maximum absorbance change of 0.2. Limited by the solubility of NMP, we used the chloroform solution at a polymer concentration of 50 mg/mL to spin on the surface of ITO glass and prepared the color-changing film. For a polymer film with an active area of 2.4 cm^2^, chronoamperometric and absorbance measurements were performed. The change in the transmittance of the EC film with time was recorded at a specified wavelength while the square wave voltage was applied. The switching time T_b_^0.9^ and T_c_^0.9^, which was defined as the time to switch between two different color states after voltage, switching reached 90% of the complete absorbance change and was determined using absorbance change plots (Figure 6).

All of these polymers showed relatively acceptable switching times (below 9 s), and their bleaching times T_b_^0.9^ were all shorter than the coloring times T_c_^0.9^, which is a common phenomenon even though there were existing counterexamples in some compound systems [24,25]. This indicates that the charge injection process was slower than extraction, which could be attributed to the congregation of the polymer film surface, which increased the potential barrier of oxidation and led to a slower dynamic process, where the path of charge extraction was faster with fewer obstacles than injection. However, other EC properties, especially cyclic stability, exhibited very different behaviors. As shown in Figure 6a, PAAS−BT showed good EC cycle stability and could still maintain 58% of the transmittance change ∆T after 450 cycles. For PAAS−TT, ∆T stabilized at about 43% after 20 cycles, and the optical contrast dropped to 80% of the maximum ∆T after 350 cycles. Correspondingly, PAAK−BT and PAAK−TT performed a poor cycling performance, except for the overall transmittance, which showed a descending trend, and the polymer films needed to achieve the maximum color contrast change after several cycles. PAAK−BT showed a maximum optical contrast of 13% during the circulation, while PAAK−TT reached a 24% optical contrast after 200 cycles; subsequently, the contrast decreased sharply after 100 cycles. In addition, due to the selection of chloroform as the processing solvent, its volatility greatly affected the polymer surface morphology, and the uneven film resulted in unstable color changes in the charge injection and extraction process. After multiple cycles, the molecular chain segments were distorted to a certain extent, and pores of a certain size were formed so that ions could better pass into and out from the interior of the film, at which moment the coloring efficiency improved. However, in this process, the microscopic structural changes also lead to the deterioration of the adhesion between the polymer film and the ITO substrate, which is eventually reflected in the diminishing of the color stability of the film.

Table 3 summarizes the EC properties of the four polymers. It is not difficult to see that the coloring efficiency (CE) of PAAS series polymers was close to 160 cm^2^/C and is much higher than that of the PAAK series polymers at only 20–30 cm^2^/C. Again, considering the J_red_/J_ox_ mentioned in the section on electrochemical properties, the PAAS series polymers had J_red_/J_ox_ values closer to 1.0 than PAAK, representing a more balanced electron injection/extraction process, which was manifested by more cycles under the square wave voltage and later optical modulation losses.

## 3. Materials and Methods

### 3.1. Materials

N, N′ -Diphenyl-*p*-phenylenediamine (95%) and N, N′-Diphenylbenzidine (98%) were provided by Energy Chemical Co., Ltd. N, N’-diphenyl-p-phenylenediamine was purified by column chromatography prior to use, and the eluent was a solution of ethyl acetate: petroleum ether at a volume ratio of 1:10. N, N’-Diphenylbenzidine was used in the reaction after recrystallization by ethyl acetate. 4-Fluorophenylsulfone (98%, Energy Chemical Co., Ltd.) was recrystallized from toluene before use. N-methyl-2-pyrrolidone (NMP) and toluene were procured from Energy Chemical Co., Ltd. and used as received. K_2_CO_3_ (Energy Chemical Co., Ltd., Shanghai, China) was vacuum-dried for 8 h before use. All synthetic and analytical procedures are described in the Supporting Information.

### 3.2. Synthesis of Polymer PAAS−BT

In a 100 mL three-neck flask with a nitrogen vent, a distillation trap, a condenser tube, and a mechanical agitator, N, N’ -diphenyl -*p*-phenylenediamine (5.21 g, 20 mmol), 4-fluorophenyl sulfone (5.08 g, 20 mmol), potassium carbonate (6.63 g, 48 mmol), N-methylpyrrolidone (41 mL, 20% solid content) and toluene (10 mL) were added as the entrainer. After stirring under the protection of a nitrogen atmosphere and heating to 150 °C to entrain the water for 4 h, the solution turned gray-green. Toluene and water were released through the distillation trap, and the solution was heated to 195 °C; the color of the solution changed from dark green to brown-yellow. After 70 h, the viscosity of the reaction system was raised. The reaction solution was slowly poured into 1000 mL of distilled water and then turned into gray-green solid strips. The obtained solids were crushed and washed three times with distilled water (1000 mL) and anhydrous ethanol (500 mL), respectively. Brown powder precipitates were collected by filtration and dried in a vacuum drying oven at 120 °C for 8 h, weighing 7.97 g (84% yield). To ensure stable performance and avoid the interference of oligomeric products, the crude polymer was successively extracted by Soxhlet extraction with acetone, THF, and chloroform. IR(KBr, cm^−1^): 3048(Ar, C-H), 1533(Ar, C=C), 1366(NAr_3_), 1192(O=S=O), 850, 780(Ar, 1,4-replaced), 710(Ar, 1-replaced); ^1^H NMR (300 MHz, CDCl_3_, δ): 7.68 (d, *J* = 8.6 Hz, 4H), 7.31 (d, *J* = 7.7 Hz, 4H), 7.21–7.08 (m, 6H), 7.06–6.93 (m, 8H).

### 3.3. Synthesis of Polymer PAAS−TT

In a 100 mL three-neck flask with a nitrogen vent, distillation trap, condenser tube, and a mechanical agitator, N, N’-diphenylbenzidine (6.73 g, 20 mmol), 4-fluorophenyl sulfone (5.08 g, 20 mmol), potassium carbonate (6.63 g, 48 mmol), N-methylpyrrolidone (47 mL, 20% solid content) and toluene (10 mL) were added as the entrainer. After stirring under the protection of a nitrogen atmosphere and heating to 150 °C to entrain the water for 4 h, the solution turned dark purple. Toluene and water were released through the distillation trap, and the solution was heated to 195 °C; the color of the solution changed from brownish yellow and dark green to brownish green. After 70 h, the viscosity of the reaction system was raised. The reaction solution was slowly poured into 1000 mL of distilled water and then turned into brown solid strips. The obtained solids were crushed and washed three times with distilled water (1000 mL) and anhydrous ethanol (500 mL), respectively. Brown powder precipitates were collected by filtration and dried in a vacuum drying oven at 120 °C for 8 h, weighing 9.06 g (82% yield). To ensure stable performance and avoid the interference of oligomeric products, the crude polymer was successively extracted by Soxhlet extraction with acetone, THF, and chloroform. IR(KBr, cm^−1^): 3046(Ar, C-H), 1528(Ar, C=C), 1366(NAr_3_), 1200(O=S=O), 856, 784(Ar, 1,4-replaced), 710(Ar, 1-replaced); ^1^H NMR (300 MHz, CDCl_3_, δ): 7.75–7.63 (m, 4H), 7.50 (d, *J* = 8.2 Hz, 4H), 7.32 (q, *J* = 7.4 Hz, 4H), 7.23–7.08 (m, 10H), 7.08–6.96 (m, 4H).

### 3.4. Synthesis of Polymer PAAK−BT

In a 100 mL three-neck flask with a nitrogen vent, a distillation trap, condenser tube, and mechanical agitator, N, N’ -diphenyl -*p*-phenylenediamine (5.21 g, 20 mmol), bis(4-fluorophenyl)-methanone (4.36 g, 20 mmol), potassium carbonate (6.63 g, 48 mmol), N-methylpyrrolidone (38 mL, 20% solid content) and toluene (10 mL) were added as the entrainer. After stirring under the protection of a nitrogen atmosphere and heating to 150 °C to entrain the water for 4 h, the solution turned orange-brown. Toluene and water were released through the distillation trap, and the solution was heated to 195 °C; the color of the solution turned dark. After 36 h, the viscosity of the reaction system was raised. The reaction solution was slowly poured into 1000 mL of distilled water and then turned into brown solid strips. The obtained solids were crushed and washed three times with distilled water (1000 mL) and anhydrous ethanol (500 mL), respectively. Brown powder precipitates were collected by filtration and dried in a vacuum drying oven at 120 °C for 8 h, weighing 7.14 g (81% yield). To ensure stable performance and avoid the interference of oligomeric products, the crude polymer was successively extracted by Soxhlet extraction with acetone, THF, and chloroform. IR(KBr, cm^−1^): 3046(Ar, C-H), 1678(C=O), 1621, 1523(Ar, C=C), 1356(NAr_3_), 1192(Ar, C-H), 939, 865, 779(Ar, 1,4-replaced), 710(Ar, 1-replaced); ^1^H NMR (300 MHz, CDCl_3_, δ): 7.74–7.64 (m, 4H), 7.32 (t, *J* = 7.7 Hz, 4H), 7.23–7.15 (m, 4H), 7.15–6.95 (m, 10H).

### 3.5. Synthesis of Polymer PAAK−TT

In a 100 mL three-neck flask with a nitrogen vent, distillation trap, condenser tube, and mechanical agitator, N, N’-diphenylbenzidine (6.73 g, 20 mmol), bis(4-fluorophenyl)-methanone (4.36 g, 20 mmol), potassium carbonate (6.63 g, 48 mmol), N-methylpyrrolidone (45 mL, 20% solid content) and toluene (10 mL) was added as the entrainer. After stirring under the protection of a nitrogen atmosphere and heating to 150 °C to entrain the water for 4 h, the solution turned gray green. Toluene and water were released through the distillation trap, and the solution was heated to 195 °C; the color of the solution turned darker. After 44 h, the viscosity of the reaction system was raised. The reaction solution was slowly poured into 1000 mL of distilled water and was then dispersed into small hard pieces in touch with the water, which broke naturally after being left overnight. The obtained solids were crushed and washed three times with distilled water (1000 mL) and anhydrous ethanol (500 mL), respectively. Brown powder precipitates were collected by filtration and dried in a vacuum drying oven at 120 °C for 8 h, weighing 7.84 g (76% yield). To ensure stable performance and avoid the interference of oligomeric products, the crude polymer was successively extracted by Soxhlet extraction with acetone, THF, and chloroform. IR(KBr, cm^−1^): 3046(Ar, C-H), 1680(C=O), 1624, 1520(Ar, C=C), 1358(NAr_3_), 1200(Ar, C-H), 942, 866, 782(Ar, 1,4-replaced), 709(Ar, 1-replaced); ^1^H NMR (300 MHz, CDCl_3_, δ): 7.71 (d, *J* = 8.3 Hz, 4H), 7.51 (d, *J* = 8.0 Hz, 4H), 7.33 (t, *J* = 7.2 Hz, 4H), 7.24–7.17 (m, 8H), 7.17–7.01 (m, 6H).

## 4. Conclusions

In summary, we synthesized several new EC polymers by a simple one-step C-N coupling reaction, which was characterized by TPA derivatives and sulfone or carbonyl groups as electron-donating groups and electron-accepting groups, respectively. The raw materials were easy to obtain, and the cost was low, which could be used for large-scale industrial production. In this series of polymers, the influence of the different electron-withdrawing capacities of the sulfone group and carbonyl group and the different phenyl or biphenyl bridging of the two TPA structures on polymer EC properties were deeply explored. The phenyl or biphenyl conjugated bridges of the two TPA structures mainly affected the oxidation potential of the polymer, while the sulfone group and the carbonyl group with different electron absorption abilities demonstrated greater influence on the energy band and cyclic stability. The optical contrast of PAAS−BT at 850 nm was up to 58% and maintained 450 cycles; other polymers also showed significant color changes even when the performance of PAAK was slightly inferior to that of PAAS, indicating that this series of materials has a broad application prospect for further research. In addition, chloroform rather than NMP was used as the solvent for film-coating in this work, which inevitably had a negative effect on the morphology of the polymer film. We believe that this material will have a better performance when the appropriate processing technology was selected for the preparation of EC devices, which still needs further exploration.

## Figures and Tables

**Table 1:** Molecular weights and thermal stabilities of PAAKs/PAASs.

	GPC Results ^a^			Thermal Stability (°C)	
Polymer	M_n_(kDa)	M_w_(kDa)	Pd	T_g_^b^	T_d5_^c^
PAAS−BT	9.9	1.5	1.55	242	446
PAAS−TT	9.3	1.2	1.25	252	443
PAAK−BT	9.4	1.1	1.18	219	530
PAAK−TT	5.7	6.5	1.15	195	526

^a^ Mn, Mw, and polydispersity (Pd) were confirmed by GPC in DMF using polystyrene as a standard reference. ^b^ Determined by DSC. ^c^ Defined as the temperature corresponding to 5% weight loss measured by TGA. Both DSC and TGA employed the same heating rate of 10 °C min^–1^ under a nitrogen atmosphere.

**Table 2:** Experimental and calculated oxidation potentials and energy levels of PAAKs/PAASs.

	Experimental							Calculated		
	Oxidation(V)				Energy Level(eV)			Energy Level(eV)		
Polymer	E_onset_	E_1/2_^ox,1^	E_1/2_^ox,2^	λ_onset_(nm)	E_g_^a^	HOMO ^b^	LUMO ^c^	HOMO	LUMO	E_g_
PAAS−BT	0.45	0.55	0.82	445	2.79	−5.25	−2.46	−5.09	−1.29	3.80
PAAS−TT	0.55	0.67		413	3.00	−5.35	−2.35	−5.08	−1.32	3.76
PAAK−BT	0.32	0.42	0.69	452	2.74	−5.12	−2.38	−4.91	−1.53	3.38
PAAK−TT	0.43	0.51		445	2.79	−5.23	−2.44	−4.93	−1.50	3.43

^a^ Energy gap = 1240/λ_onset_, with λ_onset_ calculated for the polymer film. ^b^ Experimental HOMO energy levels were calculated from the results of CV measurements and were referenced to ferrocene (4.8 eV; E_onset_ = 0.10 V). ^c^ LUMO = HOMO − E_g_.

**Table 3:** Electrochromic properties of PAAKs/PAASs.

Polymer	λ_max_(nm)	Cycles_0.8_^a^	T_c_^0.9^	T_b_^0.9^	∆OD ^b^	Q(mC/cm^2^) ^c^	CE (cm^2^/C) ^d^
PAAS−BT	600	450	6.62	2.37	0.38	2.37	160.34
PAAS−TT	695	350	6.91	5.74	0.65	4.07	159.71
PAAK−BT	633	150	8.46	8.19	0.14	6.04	23.18
PAAK−TT	705	310	7.41	7.13	0.21	6.41	32.76

^a^ Several cycles required a 20% contrast reduction. ^b^ Optical density (∆OD) = log [T_bleac_h/T_color_], where T_bleach_ and T_color_ are the maximum transmittances at the corresponding wavelength in the neutral and oxidized states, respectively. ^c^ The ejected charge Q was determined by chronoamperometry. ^d^ Coloration efficiency was calculated as CE = ∆OD/Q.

## Data Availability

The raw data supporting the conclusions of this manuscript are available to any qualified researcher.

## Supplementary Materials

The following supporting information can be downloaded, Measurments; Figures S1: ^1^H NMR spectra of the polymers PAAKs/PAASs; Figure S2: FTIR spectra of the polymers PAAKs/PAASs; Table S1: Solubility of polymers PAAKs/PAASs in different solvents; Figure S3: DSC curves of polymers PAAKs/PAASs; Figure S4: TGA curves of polymers PAAKs/PAASs; Figure S5: UV–vis absorption spectra of polymers in chloroform solution; Figure S6: Cyclic voltammograms of PAAS group and PAAK group films spin-coated on an ITO glass substrate from NMP solution at a concentration of 10 mg/mL; Figure S7: Spectroelectrochemistry of PAAS group and PAAK group films spin-casted on the ITO glass substrate from NMP solution at a concentration of 25 mg/mL.

## Sample Availability

Samples of the polymers PAAS-BT, PAAS-TT, PAAK-BT and PAAK-TT are available from the authors.
