# Supplementary material for: Poly (Aryl Amino Ketone/Sulfones) with Obvious Electrochromic Effect Prepared by One-Step Low-Cost and Facile Synthesis
Source: Molecules. 2023 Jul 9;28(14):5297. doi: 10.3390/molecules28145297 (PMC10386746; doi:10.3390/molecules28145297)
Supplement: Supplementary file 1 [file molecules-28-05297-s001.zip › molecules-2464627-SI.pdf]

Supplementary Information

PAAS/ PAAKs with obvious electrochromic effect prepared by one-step low-cost and facile synthesis

Songrui Jia[1], Zhen Xing[1], Qilin Wang [1], Shiwei Wang [2,*] and Zheng Chen[1,*] .

[1] Key of High-Performance Plastics, Ministry of Education, National & Local Joint Engineering Laboratory for Synthesis Technology of High-Performance Polymer, College of Chemistry, Jilin University, Changchun 130012, China

[2] School of Chemical Engineering, Changchun University of Technology

[*] Correspondence: chenzheng2013@jlu.edu.cn; wswjldx2004@163.com

Table of contents

1. Measurements 3

2. ^{1}H NMR spectra of polymers PAAS/PAAKs 4

3. FTIR spectra of the polymers 4

4. Solubility of polymers 5

5. DSC curves 5

6. TGA curves 6

7. UV–vis absorption spectra of solution 6

8. Cyclic voltammograms of films at lower concentration 7

9. Spectroelectrochemical tests from NMP solution 7

1. Measurments

The Fourier transform-infrared (IR) measurements (potassium bromide (KBr) pellets) were recorded in the range of 400–4000 cm^{-1} by using a Shimadzu IR Affinity-1 IR spectrometer. The nuclear magnetic resonance (NMR) spectra were recorded on a Bruker Avance 300 spectrometer (Beijing, China) at a resonant frequency of 300 MHz for 1H with deuterated chloroform ($CDCl_3$) as the solvent and tetramethylsilane as the reference. The number-average molecular weight (M_n) was determined by gel permeation chromatography (GPC) with dimethyl formamide (DMF, 1 mL/min) as the eluent. Monodispersed polystyrene was used as the molecular weight standard. Thermogravimetric analysis (TGA) and Differential scanning calorimetry (DSC) studies were carried out with a Mettler Toledo DSC821e instrument (Switzerland) at a constant heating rate of 10℃/min under nitrogen atmosphere (10 cm^3/min). TGA test was carried out under air atmosphere (10 cm^3/min) as well. The ultraviolet–visible (UV-vis) absorption spectra were obtained on a UV-2550 UV-vis spectrophotometer. Cyclic voltammetry (CV) was done on an CHI660E device (Electrochemical workstation, Shanghai, China) at room temperature by using a working electrode (indium tin oxide (ITO) polymer film area of about 8 ×30 mm^2), a reference electrode (0.01 M Ag/$AgNO_3$ in 0.1 M MeCN), and a counter electrode (platinum plate) at a sweep rate of 0.05 V/s. A 0.1M solution of tetra- butylammonium perchlorate (TBAP) in anhydrous acetonitrile was used as an electrolyte. The energy level of the highest occupied molecular orbital (HOMO) was determined from the onset oxidation $E_{onset,ox}$ based on the reference energy level of ferrocene (4.8 eV below the vacuum level) according to the following relation: HOMO= - ($E_{onset\,,\,ox}$- $E_{onset,\,ferrocene}$+ 4.8) eV. The lowest unoccupied molecular orbital (LUMO) level was calculated from the HOMO, and the value of the optical bandgap was obtained according to the relation LUMO = HOMO +E_g^{opt} (eV). The EC switching properties were measured by coupling an electrochemical workstation with an UV−vis spectrophotometer.

2. Supplementary results

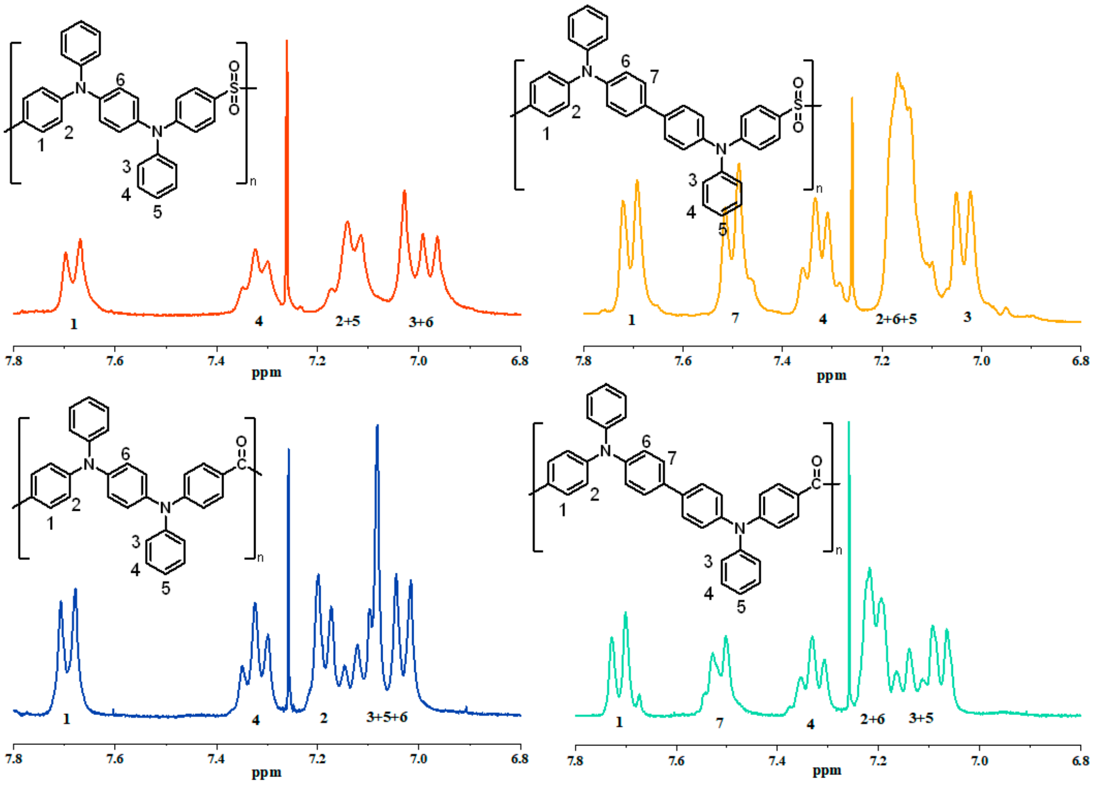

Figure S1. ^{1}H NMR spectra of the polymers PAAKs/PAASs.

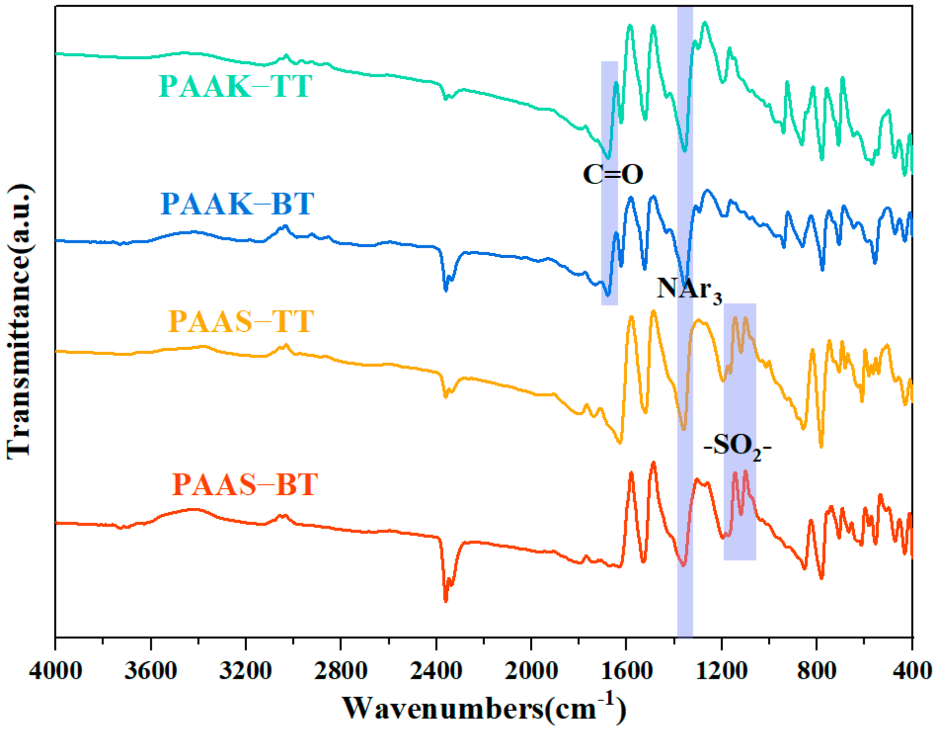

Figure S2. FTIR spectra of the polymers PAAKs/PAASs.

Table S1. Solubility of polymers PAAKs/PAASs in different solvents.

	THF	$CHCl_3$	CH_2Cl_2	DMF	DMAc	DMSO	NMP	AcN	CB
**PAAS-BT**	+	++	++	++	+	+	++	-	++
**PAAS-TT**	++	++	++	++	+	+	++	-	-
**PAAK-BT**	+	++	++	+	+	+	++	-	++
**PAAK-TT**	++	++	++	+	+	+	++	-	+

++: Soluble at room temperature; +: soluble on heating. -: insoluble on heating. Qualitative solubility was determined with as 10 mg of polymer in 1 mL of solvent.

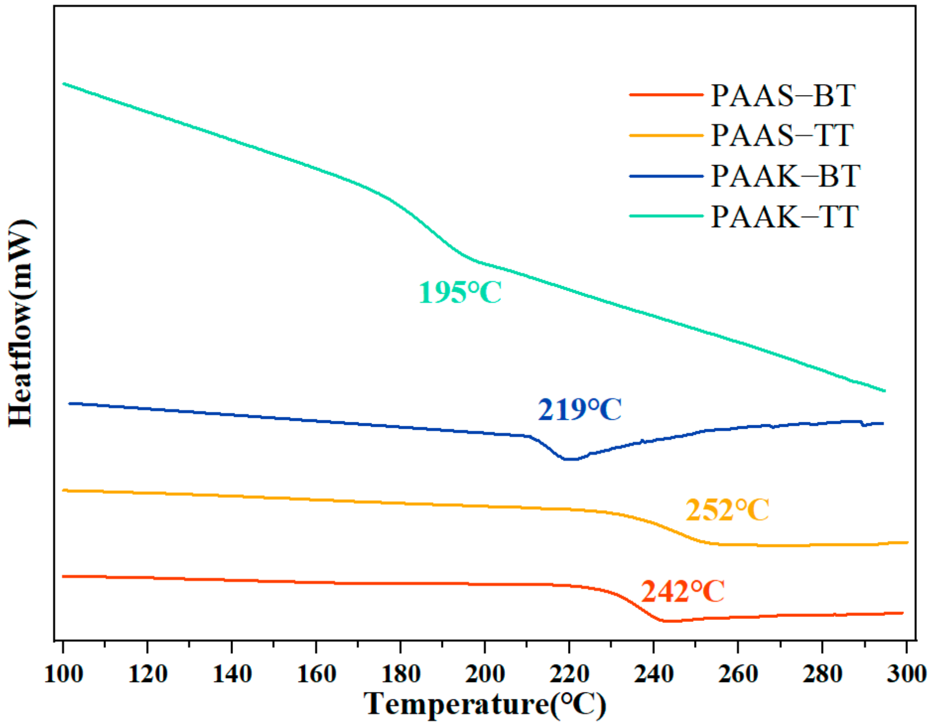

Figure S3. DSC curves of polymers PAAKs/PAASs.

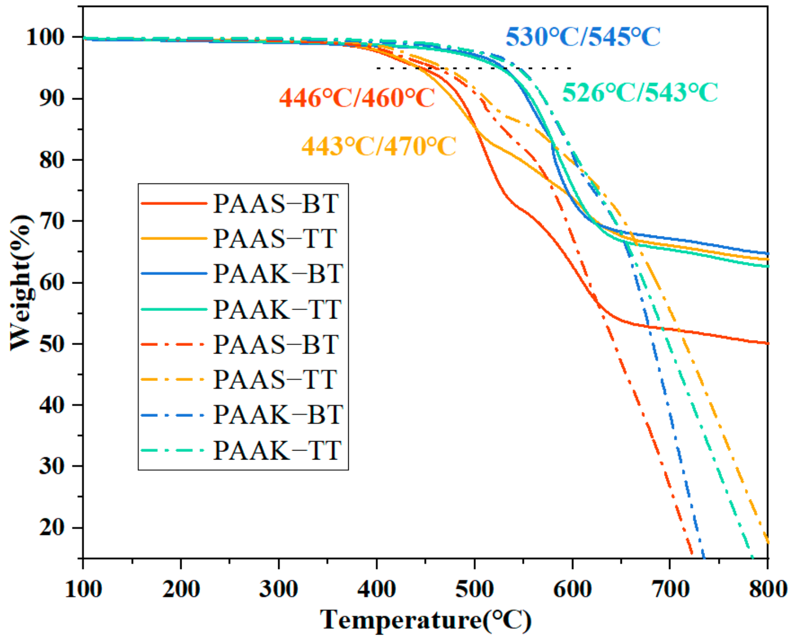

Figure S4. TGA curves of polymers PAAKs/PAASs. Solid lines mean that the tests were performed under nitrogen atmosphere while dashed lines represent air atmosphere

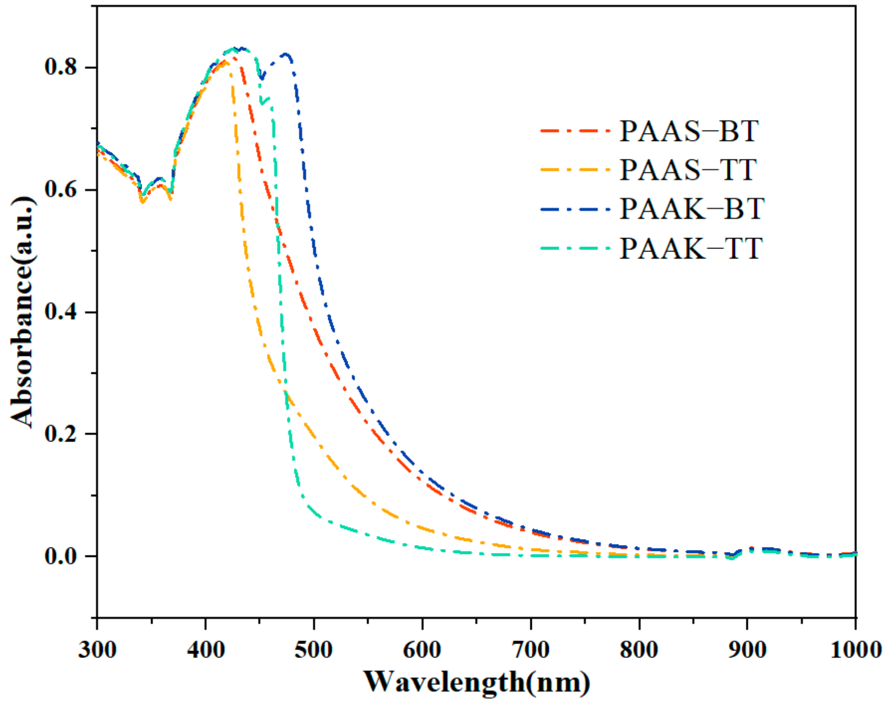

Figure S5. UV–vis absorption spectra of polymers in chloroform solution at a concentration of 10^{-5} M.

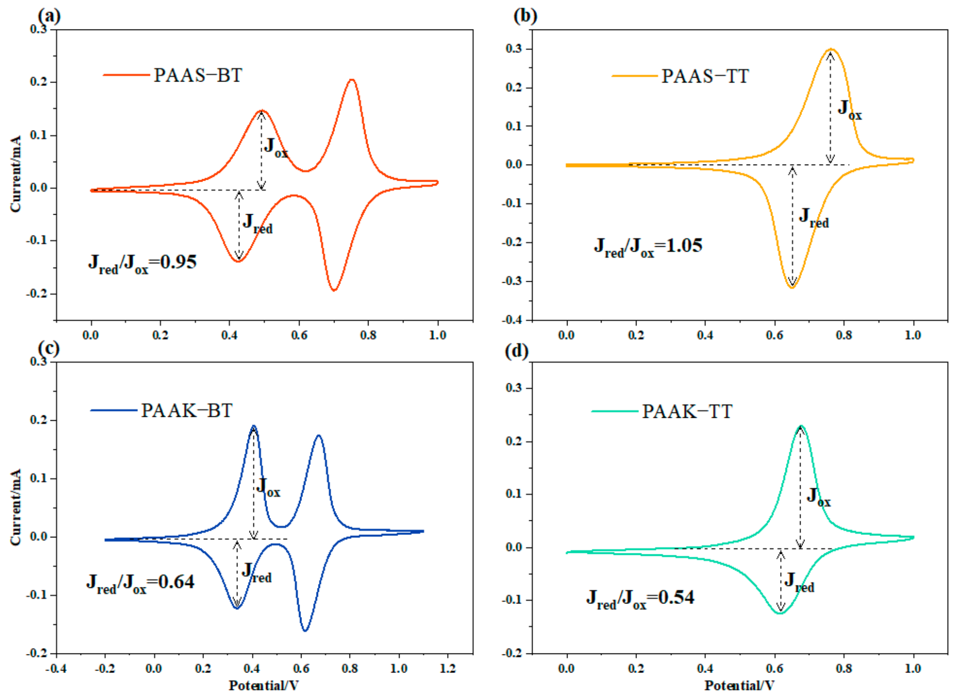

Figure S6. Cyclic voltammograms of (a, b) PAAS group and (c, d) PAAK group films spin-coated on an ITO glass substrate from NMP solution at a concentration of 10 mg/mL (Scan rate: 50 mV/s).

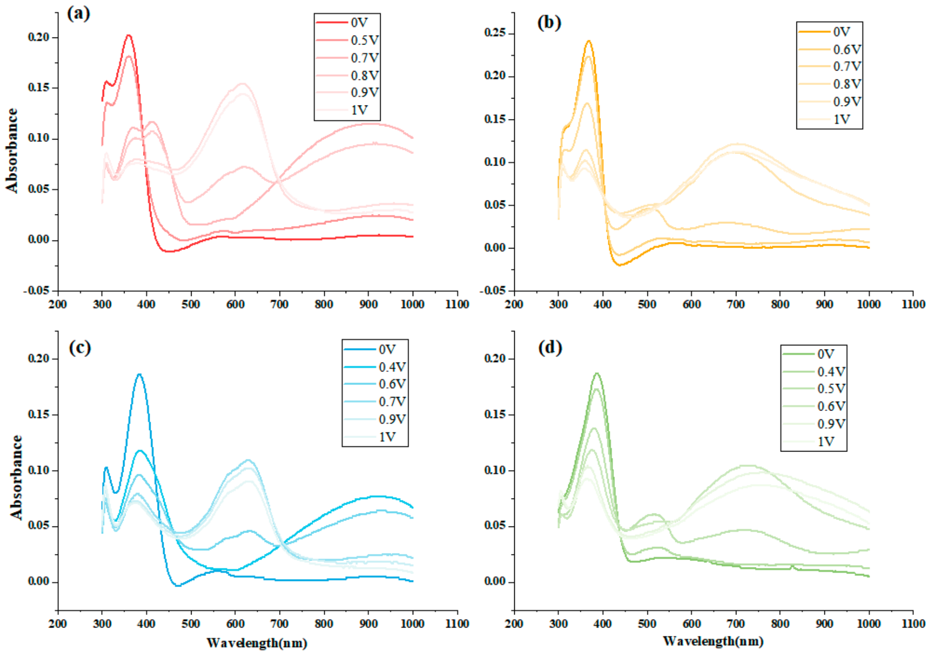

Figure S7. Spectroelectrochemistry of (a, b) PAAS group and (c, d) PAAK group films spin-casted on the ITO glass substrate from NMP solution at a concentration of 25 mg/mL.
